# P-1457. Vaccination trends for tetanus, hepatitis A, and hepatitis B in individuals with a substance use disorder, hospitalized at a United States integrated health care system, 2010 - 2023

**DOI:** 10.1093/ofid/ofaf695.1643

**Published:** 2026-01-11

**Authors:** Jennifer H Ku, Cheyne Hoke, Yuqian M Gu, Hung Fu Tseng, Yi Luo, Rulin C Hechter, Bradley Ackerson, Cara D Varley

**Affiliations:** Kaiser Permanente Hawaii, Honolulu, HI; Kaiser Permanente Southern California, Pasadena, California; Kaiser Permanente Southern California, Pasadena, California; Kaiser Permanente Southern California, Pasadena, California; Kaiser Permanente Southern California, Pasadena, California; Kaiser Permanente Southern California Department of Research and Evaluation, Los Angeles, California; Kaiser Permanente Southern California, Pasadena, California; Oregon Health & Science University, Portland, OR

## Abstract

**Background:**

Individuals with substance use disorders (SUD) are at high risk for hepatitis A virus (HAV), hepatitis B virus (HBV), and tetanus infections. Hospitalizations are frequent for this population and may represent opportunities for preventive care, but data on vaccination trends are limited in this setting.

**Figure 1.**
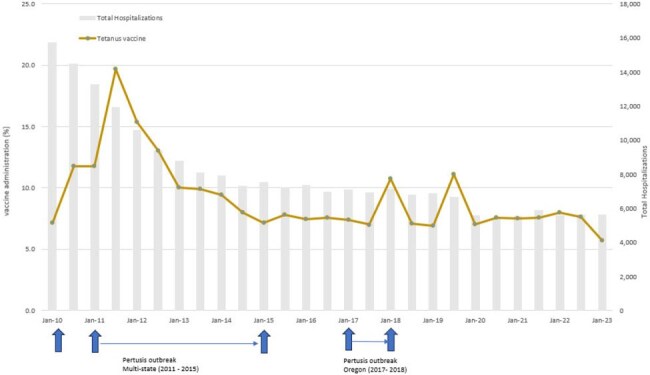
Tetanus vaccine administration during hospitalization or within 90 days of discharge among individuals with a substance use disorder (2010 – 2023)

**Figure 2.**
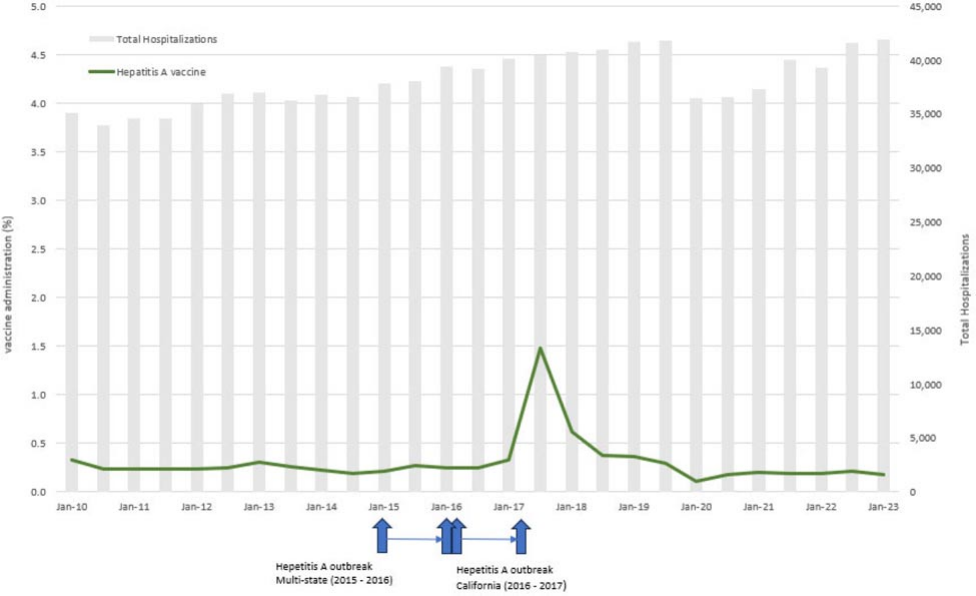
Hepatitis A vaccine administration during hospitalization or within 90 days of discharge among individuals with a substance use disorder (2010 – 2023)

**Methods:**

Using electronic health records (EHR) at Kaiser Permanente Southern California, we identified adults ≥18 years of age, with a SUD diagnosis within 2 years prior to the first hospitalization between 01/2010 and 06/2023 (study period), who were at risk for tetanus (no history of tetanus vaccination), HAV, or HBV (no history of vaccination/infection/immunity). We examined vaccination rates during hospitalizations or within 90 days of discharge during the study period. We divided the study period into 6-month intervals. We calculated vaccination rate for each 6-month interval by dividing the number of vaccinated individuals by the number of hospitalized at-risk individuals for each 6-month interval. If >1 hospitalizations occurred within a 6-month interval, only the first hospitalization was included.

**Figure 3.**
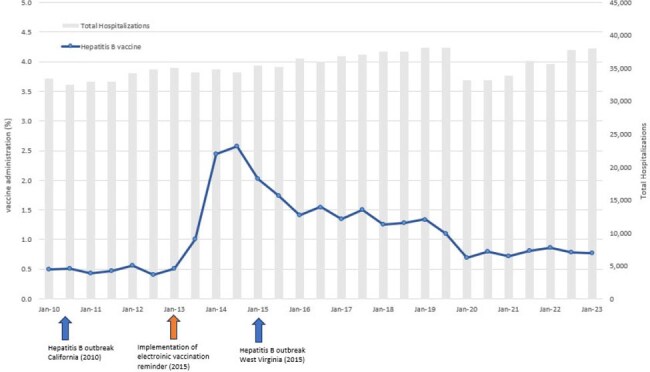
Hepatitis B vaccine administration during hospitalization or within 90 days of discharge among individuals with a substance use disorder (2010 – 2023)

**Results:**

We identified 97,240 (mean age 48 years [S.D. 17], 39% female, 47% Non-Hispanic White), 377,774 (mean age 50 years [S.D. 17], 42% female, 47% Non-Hispanic White), and 354,073 (mean age 52 years [S.D. 17], 41% female, 48% Non-Hispanic White) individuals at risk for tetanus, HAV and HBV, respectively. Overall, tetanus vaccination rate was 9.7% (among 217,530 hospitalizations among at-risk individuals) during the study period (Figure 1); vaccination rate was 0.3% (among 1,031,226 hospitalizations among at-risk individuals) for HAV (Figure 2) and 1.1% (among 955,689 hospitalizations among at-risk individuals) for HBV (Figure 3). Vaccination rates remained largely unchanged during the study period, with several peaks coinciding with local and national pertussis, HAV and HBV outbreaks, as well as implementation of EHR vaccine reminders for HBV.

**Conclusion:**

Vaccination rates for tetanus, HAV and HBV for individuals with SUD at hospitalization or subsequent follow-up were low during 2010 - 2023, with peaks coinciding with outbreaks. Our results point to missed opportunities for vaccination in this high-risk population.

**Disclosures:**

Jennifer H. Ku, PhD MPH, AstraZeneca: Grant/Research Support|GSK: Grant/Research Support Yuqian M. Gu, MS, GSK: Grant/Research Support Hung Fu Tseng, PhD MPH, AstraZeneca: Grant/Research Support|GlaxoSmithKline: Grant/Research Support|Moderna: Grant/Research Support Yi Luo, PhD, AstraZeneca: Grant/Research Support|GlaxoSmithKline: Grant/Research Support|Moderna: Grant/Research Support Rulin C. Hechter, MD, PhD, MS, Opioid Post Marketing Requirements Consortium (OPC): Grant/Research Support Bradley Ackerson, MD, AstraZeneca: Grant paid to KPSC for work unrelated to this study.|Dynavax: Grant paid to KPSC for work unrelated to this study.|F2G: Grant paid to KPSC for work unrelated to this study.|GSK: Grant paid to KPSC for work unrelated to this study.|Moderna: Grant paid to KPSC for work unrelated to this study.|Pfizer: Grant paid to KPSC for work unrelated to this study.

